# Peroneal artery injury potential due to different syndesmosis screw placement options: a simulation study with lower extremity computed tomography angiography

**DOI:** 10.1007/s00402-024-05258-w

**Published:** 2024-03-16

**Authors:** Kürşad Aytekin, İsmet Miraç Çakır, Merve Nur Taşdemir, Orhan Balta

**Affiliations:** 1 School of Medicine, Department of Orthopedics and Traumatology and Department of Anatomy, University of Giresun, Giresun, Turkey; 2 School of Medicine, Department of Radiology, University of Giresun, Giresun, Turkey; 3 Department of Orthopaedics and Traumatology, Gaziosmanpasa University Hospital, Kaleardı District Muhittin Fisunoglu Street, 60100 Tokat, Turkey

**Keywords:** Syndesmosis screw, Peroneal artery, Iatrogenic injury, Computed tomography angiography

## Abstract

**Introduction:**

The aim of this study is to assess the risk of peroneal artery injury of hardware placement at the fixation of syndesmotic injuries.

**Materials and methods:**

The lower extremity computed tomography angiography was used to design the study. The syndesmosis screw placement range was simulated every 0.5 cm, from 0.5 to 5 cm proximal to the ankle joint. The screw axes were drawn as 20°, 30° or individual angle according to the femoral epicondylar axis. The proximity between the screw axis and the peroneal artery was measured in millimeters. Potential peroneal artery injury was noted if the distance between the peroneal artery to the axis of the simulated screw was within the outer shaft radius of the simulated screw. The Pearson chi-square test was used and a *p*-value < 0.05 was considered significant.

**Results:**

The potential for injury to the peroneal artery increased as the syndesmosis screw level rose proximally from the ankle joint level or as the diameter of the syndesmosis screw increasds. In terms of syndesmosis screw trajection, the lowest risk of injury was observed with the syndesmosis screw angle of 20°. Simulations with a screw diameter of 3.5 mm exhibited the least potential for peroneal artery injury.

**Conclusion:**

Thanks to this radiological anatomy simulation study, we believe that we have increased the awareness of the peroneal artery potential in syndesmosis screw application. Each syndesmosis screw placement option may have different potential for injury to the peroneal artery. To decrease the peroneal artery injury potential, we recommend the followings. If individual syndesmosis screw angle trajection can be measured, place the screw 1.5 cm proximal to the ankle joint using a 3.5 mm screw shaft. If not, fix it with 30° trajection regardless of the screw diameter at the same level. If the most important issue is the peroneal artery circulation, use the screw level up to 1 cm proximal to the ankle joint regardless of the screw angle trajection and screw diameter.

## Introduction

The syndesmosis ligament contributes to the stability of the ankle [1]. Syndesmosis ligament injuries can be seen in approximately 25% of all ankle injuries [2]. In case of syndesmosis ligament injury, syndesmosis screws are used to aid in the healing of ligaments at appropriate positions [3]. Different syndesmosis screw placement techniques are used for the fixation of ankle syndesmosis injuries. Syndesmotic fixation techniques may exhibit variations with regard to screw proximity to the ankle joint, trajectory of the screw and diameter of the screw [1, 4–10]. Although studies in the literature usually focus on the biomechanics of screw placement and techniques to minimize syndesmosis malreduction, few studies investigate the potential of peroneal artery injury [1, 11, 12]. But these few studies examined only the proximity distance between the syndesmosis screw and the peroneal artery via only one or two syndesmotic fixation techniques due to the nature of cadaver studies [11].

Arteria fibularis is close to the syndesmosis ligament and contributes to the nutrition of the syndesmosis ligament at the ankle [12]. The peroneal artery provides the majority of the anterior ligamentous vascular supply to the tibiofibular syndesmosis and contributes to the blood supply of the foot especially the talus bone through the dorsalis pedis artery [2, 12, 13]. Insufficient vascular supply to an area of ligamentous injury, specifically the tibiofibular syndesmosis, may lead to delayed healing and increased rates of complications [12]. Sometimes the peroneal artery continues as the dorsalis pedis artery [14]. It is known that the peroneal artery is effective in increasing blood pressure in the circulation of the toes [15].

In some radiological simulation studies,MRI or computed tomography angiography images have been used to minimize iatrogenic injury risks to soft tissue that may occur during surgical approaches to the ankle or different regions. Hamada et al. simulated the suture-button technique using magnetic resonance images and investigated the iatrogenic injury risk of the saphenous vein and nerve [16]. Mousa et al. examined the peroneal artery injury risk for the posterolateral approach to distal tibia via computed tomography angiography [17]. Besides the studies performed at ankle region, an anatomical relationship between simulated S1 sacroiliac screws’ entrance points and superior gluteal artery has been investigated by computed tomography angiography [18]. In the present study, we aim to investigate the potential for injury to the peroneal artery depending on syndesmotic screw fixation in relation to screw position, diameter and trajectory.

## Materials and methods

This retrospectively designed study was performed in Giresun university training hospital. The lower extremity computed tomography angiography studies performed between January 2017 and January 2023 were used to examine the lower extremity arteries. At our hospital, the radiology department captures images of both lower extremities during lower extremity computed tomography angiography. The inclusion criteria were; being older than 18 years, observable arteria fibularis, arteria tibialis anterior and arteria tibialis posterior at ankle level to be sure the circulation of the arteria fibularis is working, observable epicondylar axis of the distal femur to be able to precisely measure the simulated syndesmosis screw angle. The exclusion criteria were; images that were not appropriate for evaluation, individuals who have undergone lower extremity arthroplasty surgery, those with previous lower extremity fractures, those with previous syndesmosis injury, those with congenital hip dysplasia or other lower extremity congenital diseases.

### Image acquisition

Multidetector CT protocol. All images were obtained with a 128-slice MDCT system (GE Revolution EVO 128; GE Healthcare, Milwaukee, WI, USA). The patients were placed in the supine position with their feet first in the gantry. All lower extremities were included in all cases and image parameters were as follows: section thickness: 1.25 mm, tube voltage: 100–120 kV, gantry rotation: 0.6 s, and pitch value: 1.0 mm. The contrast agent bolus was applied with an automatic injector pump. The amount of contrast agent Omnipaque 350® (Iohexol, GE Healthcare, Milwaukee, WI, USA) used in all applications was between 110 and 150 mL, depending on the body mass of the patient. The contrast agent administration rate was 4 mL/s.

### Image analysis

All CT images were reviewed in a randomized order by a radiology specialist and an orthopedics & traumatology specialist with the use of a dedicated computed workstation.

### Determination of the height of placement for simulated syndesmosis screw

**Fig. 1:**
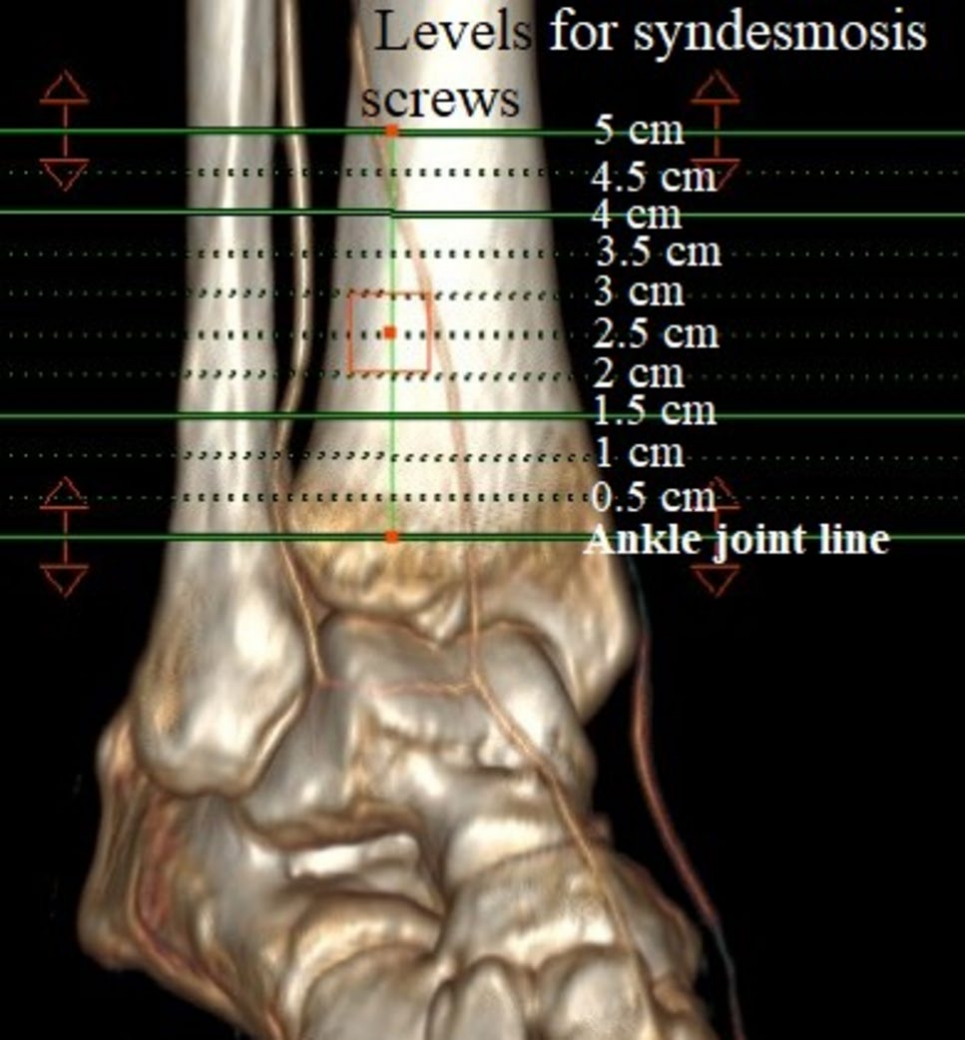
The 10 different placement levels of the simulated syndesmosis screws. Please note that every level has three different syndesmosis screw angle option, each with three different screw shaft diameter option

The investigated syndesmosis screw placement proximal to the ankle joint ranged from 0.5 to 5 cm [2, 4, 9, 19]. Therefore, we simulated the syndesmosis screws from every 0.5 cm proximal to the ankle joint. The simulated syndesmosis screw heights were as follows: 0.5, 1, 1.5, 2, 2.5, 3, 3.5, 4, 4.5 and 5 cm proximal to the ankle joint (Fig. 1).

### Determination of syndesmosis screw angle degrees

**Fig. 2:**
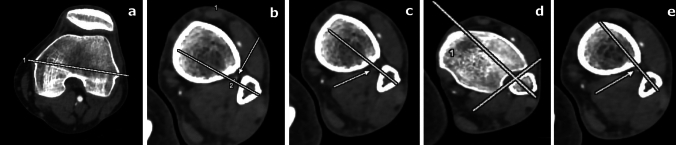
The necessary axes for the syndesmosis screw placement and the relation of the axes and peroneal artery. The arrows show the peroneal artery. At the **b**, **c** and **e**, the three different diameter screw options have potential for damage to the peroneal artery. **a** The transepicondylar femoral axes. **b** The axis of the 20° syndesmosis screw angle. The level is 2.5 cm proximal to the ankle joint line. The closest distance of axis to the peroneal artery is 0.9 mm. **c** The axis of the 30° syndesmosis screw angle. The level is 2.5 cm proximal to the ankle joint line. The figure points out the overlapping of the simulated syndesmosis screw axis and peroneal artery. **d** The individual syndesmosis screw angle perpendicular to the incisura fibularis. The level is 0.5 cm proximal to the ankle joint line. **e** The individual syndesmosis screw angle parallel to the interosseous ligament above 3 cm to the ankle joint. Please note the proximity of the peroneal artery to the syndesmosis screw axis. The syndesmosis screw axis passes tangent to the peroneal artery

In the literature, there are different suggestions about the syndesmosis screw fixation angle from posterior to anterior. These suggested angles are usually 20°, 30° or perpendicular to the fibular notch [7, 8, 10]. The angles were defined as the angle between the femoral epicondylar axis line (Fig. 2a) and the simulated syndesmosis screw axis line. We simulated the syndesmosis screw placement angles as 20°, 30° (Fig. 2b, c). A third syndesmosis screw angle, which can vary individually at different levels for the placement of the simulated syndesmosis screw, was also used. This angle known as individual syndesmosis screw angle is determined as follows [8, 20]. If the fibular notch is visible on CT images, a perpendicular line is drawn to the incisura fibularis (Fig. 2d). If not, a parallel line is drawn to the interosseous ligament (Fig. 2e). The angle between the femoral epicondylar axis line and the individually drawn simulated syndesmosis screw axis line is defined as “individual syndesmosis screw angle”.

### Defining the proximity distance between the simulated syndesmosis screw axis and arteria fibularis

At each of the 10 different levels of simulated syndesmosis screw placements, the three simulated syndesmosis screw axes were examined one by one. In each ankle, a total of 30 simulated syndesmosis screw axes were investigated using computed tomography angiography. The arteria fibularis was detected at each of computed tomography angiographies at the ankle level. The closest distance of the simulated syndesmosis screw axis to the peroneal arery was measured in millimeters (Fig. 2b, c, e).

### Deciding the injury risk of arteria fibularis due to the simulated syndesmosis screw

In the literature, the suggested screw diameter for the fixation of the syndesmosis injury varies from 3.5 to 4.5 mm [2]. Accordingly, we simulated the screw shaft diameter as 3.5, 4 and 4.5 mm. We assumed that the arteria fibularis, within the diameter of the simulated syndesmosis screw, may be injured in surgery during the syndesmosis screw placement. Therefore, the radius of the simulated screw was considered clinically to have the potential to damage the arteria fibularis, if the proximity distance is equal or less than the radius (Fig. 2b, c, e). On account of this, if the distance between the simulated syndesmosis screw axis and the arteria fibularis was ≤ 1.75 mm for 3.5 mm screws, ≤ 2 mm for 4 mm screws, and ≤ 2.25 mm for 4.5 mm screws, it was assumed that the arteria fibularis could be at the risk of injury (Fig. 2b, c, e).

### Statistics

Data were expressed as mean ± standard deviation or frequency and percent. The Pearson chi-square test was used to compare the categorical data between/among groups. Categorical variables were presented as a count and percentage. A *p*-value < 0.05 was considered significant. Statistical analyses were performed using SPSS 22 (IBM SPSS Statistics 22, SPSS inc., an IBM Co., Somers, NY).

## Results

One hundred computerized tomography angiography images were investigated. At the ankle region, 10 different syndesmosis screw levels, with three different syndesmosis screw angles and three different syndesmosis screw diameters were simulated. According to these simulations, 90 simulated syndesmosis screws were evaluated at each ankle. At the end, a total of 9000 simulated syndesmosis screws were investigated for their potential to cause injury to the peroneal artery. The injury potential of the peroneal artery according to different syndesmosis screw levels, different syndesmosis screw trajection angles and different syndesmosis screw diameters are given in Table 1. The peroneal artery injury potential was present for 3501 (38.9%) simulated syndesmosis screws. It was found that the individual syndesmosis screw angles increased as the syndesmosis screw level rose proximally from the ankle joint. The potential risk of injury to the peroneal artery elevated as the syndesmosis screw level rose more proximal to the ankle joint level or the diameter of the syndesmosis screw increased. When evaluated in terms of syndesmosis screw trajection, the lowest risk of injury was observed with the individual syndesmosis screw angles of 30° and 20°, respectively, at the screw levels up to 2.5 cm proximal to the ankle joint. For the 3 cm and more proximal to the ankle joint levels, the risk of peroneal artery injury rose with an increase in the syndesmosis screw angle.

**Table 1:** Injury potential of the peroneal artery according to the simulations of the different syndesmosis screw levels above the ankle joint, the different syndesmosis screw angles and the different diameters of the screws

Level of simulated syndesmosis screw above the ankle joint	Syndesmosis screw trajection angle									
	20°			30°			Individual syndesmosis angle			
	Screw diameter			Screw diameter			Screw diameter			Angle degree (mean ± SD)
	3.5 mm	4 mm	4.5 mm	3.5 mm	4 mm	4.5 mm	3.5 mm	4 mm	4.5 mm	
5 cm	43% (a)	47% (a)	54% (a)	59%(ab)	60%(ab)	65% (ab)	85 (a)	87% (a)	87% (a)	41.970 ± 7.25
4.5 cm	39% (a)	46% (a)	50% (a)	57% (ab)	62% (ab)	64% (ab)	87% (a)	87% (a)	89% (a)	40.790 ± 7.07
4 cm	40% (a)	47% (a)	51% (a)	61% (b)	62% (ab)	66% (ab)	82% (a)	85% (a)	86% (a)	39.490 ± 7.00
3.5 cm	45% (a)	51% (a)	54% (a)	60% (ab)	65% (b)	67% (b)	80% (a)	81% (a)	84% (a)	38.450 ± 6.67
3 cm	42% (a)	51% (a)	51% (a)	51% (ab)	62% (ab)	65% (ab)	70% (a)	73% (a)	76% (a)	36.930 ± 6.42
2.5 cm	42% (a)	43% (a)	47% (a)	37% (ac)	41% (ac)	44% (ac)	36% (b)	41% (b)	45% (b)	35.910 ± 6.32
2 cm	32% (a)	33% (a)	36% (a)	17% (cd)	22% (c)	24% (c)	14% (c)	14% (c)	15% (c)	34.480 ± 6.37
1.5 cm	7% (b)	8% (b)	10% (b)	4% (de)	4% (d)	4% (d)	1% (d)	2% (cd)	2% (d)	32.990 ± 6.84
1 cm	0% (b)	0% (b)	0%(b)	0% (e)	0% (d)	0% (d)	0% (d)	0% (d)	0% (d)	31.120 ± 6.72
0.5 cm	0% (b)	0%(b)	0% (b)	0% (e)	0% (d)	0% (d)	0% (d)	0% (d)	0% (d)	29.420 ± 6.61
*p*	< 0.001	< 0.001	< 0.001	< 0.001	< 0.001	< 0.001	< 0.001	< 0.001	< 0.001	

The results of injury potential to the peroneal artery for each probability according to the different options are given as a percentage. At the last column, the mean individual syndesmosis screw angles are given according to the level of syndesmosis screws

*Pearson chi-square test* was used. a, b, c, d, e: the common letter in the column denotes statistical insignificance

Table 2 shows the potential peroneal artery injury based on the simulated screw's level above the joint, trajectory and diameter. The lower the level of syndesmosis screw, the lower potential for peroneal artery injury. In the comparison between simulated syndesmosis screw trajections, the lowest peroneal artery injury potential was found at the screw trajection angle of 20°. When simulated syndesmosis screw diameters were compared, the peroneal artery injury potential was found to be lower at 3.5 and 4 mm compared to 4.5 mm.

**Table 2:** The peroneal artery injury potential for each variable separately

Variables	Total simulated screws	The simulated screws those have potential to damage to peroneal artery *n* (%)	*p*
Simulated syndesmosis screw level			
5 cm	900	587 (65.2%) (a)	< 0.001
4.5 cm	900	581 (64.6%) (a)	
4 cm	900	580 (64.4%) (a)	
3.5 cm	900	587 (65.2%) (a)	
3 cm	900	541 (60.1%) (a)	
2.5 cm	900	376 (41.8%) (b)	
2 cm	900	207 (23%) (c)	
1.5 cm	900	42 (4.7%) (d)	
1 cm	900	0 (0%) (e)	
0.5 cm	900	0 (0%) (e)	
Simulated syndesmosis screw trajection			
20°	3000	969(32.3%) (a)	< 0.001
30°	3000	1123(37.4%) (b)	
Individual syndesmosis angle	3000	1409(47) (c)	
Simulated syndesmosis screw diameter			
3.5 mm	3000	1091(36.4%) (a)	< 0.001
4 mm	3000	1174(39.1%) (ab)	
4.5 mm	3000	1236(41.2%) (b)	

*Pearson chi-square test* was used. a, b, c, d, e: the common letter in the column denotes statistical insignificance

## Discussion

The fixation of syndesmotic injuries is a critical aspect of ankle joint stabilization, aiming to restore stability and promote proper healing [3]. However, the risk of peroneal artery injury associated with hardware placement in syndesmotic fixation remains a concern. In this study, we investigated the potential for peroneal artery injury with different syndesmosis screw placement techniques and provided recommendations to minimize this risk.

In this study, the potential for peroneal artery injury during surgical fixation of the syndesmosis joint was investigated using lower extremity computed tomography angiography simulation. The overall injury potential of the simulated screws was found to be 38.9%, with the injury potential increasing as the screw level rose proximally to the ankle joint, the screw trajectory angle increased, and the screw shaft diameter increased. These results align with previous studies indicating that screw placement location and size can influence the proximity to vital structures, including the peroneal artery [1, 11, 12].

Previous cadaveric studies have also investigated the risk of peroneal artery injury with limited syndesmosis screw options [1, 9, 11, 21]. However, this simulation study explored 90 different syndesmosis screw technique options on each ankle angiography CT, providing a comprehensive analysis of the potential risk of peroneal artery injury.

Different perspectives and recommendations exist regarding syndesmosis screw placement techniques [2, 21, 22]. Some studies focus on stability, suggesting screw placement 2 cm above the ankle joint, while others consider the risk of injury to the hyaline cartilage and recommend screw placement at least 12 mm above the joint level [2, 22]. The present study found no peroneal artery injury risk at 0.5 and 1 cm proximal to the ankle joint. Placement of the syndesmosis screw up to two cm proximal to joint level may have a low risk of arteria damage but it must be avoided to save cartilage of the distal tibiofibular joint. Furthermore, surgeons are not free to choose the angulation of the screw due to fracture lines and plate positioning.

Notably, our study demonstrated that the lowest risk of peroneal artery injury was observed with individual syndesmosis screw angles, specifically at screw levels up to 2.5 cm proximal to the ankle joint. For screw levels 3 cm or more proximal to the ankle joint, we recommend a 20° trajectory for screw placement. These recommendations provide valuable guidance for clinicians in selecting appropriate screw angles and levels to minimize the risk of peroneal artery injury during syndesmotic fixation.

Moreover, the importance of preoperative imaging, particularly lower extremity computed tomography angiography (CTA), was highlighted in our study. When CTA is available, we recommend placing the screw 1.5 cm proximal to the ankle joint using a 3.5 mm screw with an individual syndesmosis screw angle trajectory. This approach allows for precise preoperative planning and minimizes the risk of peroneal artery injury. However, in cases where CTA is not feasible, fixation with a 30° trajectory is recommended irrespective of the screw diameter at the same level.

The peroneal artery is crucial for the vascular supply of the foot and ankle. The peroneal artery provides the majority of the anterior ligamentous vascular supply and contributes to the blood supply of the foot and talus bone through the dorsalis pedis artery [2, 11]. Although, we do not commonly see acute complications as bleeding and circulatory disorders in the daily clinical practice, some complications due to the injury of the peroneal artery have been documented. Ward et al. presented a compartment syndrome of the lower extremity following an ankle injury involving peroneal artery damage [23]. Iatrogenic injury to the peroneal artery due to the syndesmosis screw for syndesmotic fixation may cause lower extremity compartment syndrome [11]. Although the certain benefit of surgical fixation in syndesmosis injury is known, adverse effects can be seen after syndesmosis fixation and the certain reasons are unclear [24]. At the same time, the reason for prolonged recovery time after syndesmotic ligament injury is unclear [12]. In a clinical study, complex regional pain syndrome was reported in 17% of patients after ankle surgery with high incidence of anxiety or depression, however, no statistically significant anatomic reason could be found [19]. Besides, the talus predisposition to avascular necrosis and the lack of vascular redundancy make each vascular contribution vitally important [25]. Especially for multitrauma patients including those with syndesmosis rupture and talus fracture, the peroneal artery survival may be more important [26]. Another importance of the peroneal artery is the usage of flaps to cover soft tissue defects [25]. Reverse peroneal artery flaps are crucial especially for the soft tissue defects at ankle or foot regions [25]. Thus, special attention should be given to the double fixation of the syndesmosis. Considering the significance of peroneal artery preservation, it is noteworthy that syndesmosis screw placement at 0.5 or 1 cm proximal to the ankle joint, regardless of the screw angle trajectory or diameter, can be employed to prioritize peroneal artery circulation. However, it is crucial to weigh this consideration against other factors, such as the overall stability and alignment of the syndesmotic complex.

Although our study provides valuable insights into reducing the risk of peroneal artery injury during syndesmotic fixation, further prospective radiological and clinical studies are necessary. Doppler ultrasonography and advanced imaging techniques can offer additional assessment of accurate iatrogenic peroneal artery injury, enabling a more comprehensive evaluation of the recommended syndesmosis screw fixation techniques.

It is important to note that this study has limitations, including its retrospective nature, the use of images from a single location and the lack of cadaveric evaluation or post-surgery assessment using Doppler ultrasonography. Utility of this study is inherently limited due to its simulated nature. Although it increases awareness of peroneal artery injury, it is hard to draw clinic conclusions. Another limitation of the study is the uncertainity of the position of the peroneal artery due to trauma. What effect does clamping the syndesmosis, or alternatively injury to the syndesmosis, have on peroneal artery position? Could the peroneal artery be closer or farther away to the screw diameter at these levels and positions? Unfortunately, these could not be assessed in this study.

In conclusion, our study underscores the importance of careful screw placement in syndesmotic fixation to mitigate the risk of peroneal artery injury. Although definitive clinical results cannot be given due to the nature of this radiological anatomy study, we believe that we have increased the awareness of the peroneal artery in syndesmosis screw application. Each syndesmosis screw placement option has different injury potential to the peroneal artery.

We provide practical recommendations based on our findings and emphasize the significance of preoperative imaging for precise planning. Implementing these recommendations can enhance patient safety and optimize outcomes in the surgical management of syndesmotic injuries. We recommend the following syndesmosis screw placement options to minimize the peroneal artery injury potential; (1) use a 3.5 cm shaft diameter at 1.5 cm proximal to the ankle joint level with individual syndesmosis screw angle trajection if it can be measured; if not, use 30° trajection with any shaft diameter. (2) If circulation is crucial for the foot or ankle, we recommend placing of the syndesmosis screw up to 1 cm proximal to the ankle joint, regardless of the screw angle trajection and screw diameter. (3) For the syndesmosis screw levels up to 2.5 cm proximal to the ankle joint, we recommend individual syndesmosis screw angles if they can be measured; if not, we recommend 30° screw angle trajection. (4) For levels of 3 cm or more proximal to the ankle joint, we recommend a screw angle trajection of 20°. (5) In terms of screw shaft diameter, the 3.5 mm shaft diameter reduces the risk of peroneal artery injury. Further prospective radiological and clinical studies are needed, including doppler ultrasonography for thoroughly examining the accurate iatrogenic peroneal artery injury, to suggest a more appropriate syndesmosis screw fixation technique.

## Data Availability

The risk of peroneal artery injury is statistically lowest in the first 1.5 cm, but there is no risk of injury in the first 1 cm.
